# Characterization of Surface Modification of 347 Stainless Steel upon Shot Peening

**DOI:** 10.1155/2017/2189614

**Published:** 2017-12-12

**Authors:** Kejian Li, Qiang Zheng, Chunhong Li, Bin Shao, Donglin Guo, Dengming Chen, Jianchun Sun, Jiling Dong, Pengjun Cao, Keesam Shin

**Affiliations:** ^1^School of Metallurgy and Materials Engineering, Chongqing University of Science & Technology, Chongqing 401331, China; ^2^The Center of Material Analysis and Testing, Chongqing University of Science & Technology, Chongqing 401331, China; ^3^School of Nano & Advanced Materials Engineering, Changwon National University, Changwon 51140, Republic of Korea

## Abstract

Plastic deformations, such as those obtained by shot peening on specimen surface, are an efficient way to improve the mechanical behavior of metals. Generally, scanning electron microscopy (SEM) and electron backscattered diffraction (EBSD) are commonly used to observe the complex microstructural evolutions, such as grain refinement and phase transformation, induced by the surface treatment. In this work, the microstructure of 347 stainless steel, after ultrasonic shot peening (USP) treatments, was investigated. SEM, EBSD, transmission electron microscopy, and X-ray diffraction were used to observe the microstructural evolutions, such as grain refinement and phase transformation. Deformation depth after the USP treatment was about 200 *μ*m. Grain size on the treated surface layer was about 100 nm, with two phases: austenite and *α*′-martensite. The percentages of the austenite and *α*′-martensite phases were 54% and 46%, respectively, which constitute an exact expression of the degree of plastic deformation on austenitic stainless steel.

## 1. Introduction

Austenitic 347 stainless steel exhibits extremely good ductility and is used in applications requiring good formability, such as the formation of furnace tubes in petrochemical industries, mainly because of its corrosion resistance and mechanical strength. This kind of stainless steel is stabilized with the addition of niobium (Nb), thus preventing sensitization-related corrosion failures as well as operational and maintenance errors that may result in premature failure [1]. After annealing, a soft austenitic structure is obtained, which will be converted into *α*′-martensite during subsequent operations, such as cold working and refrigeration treatments. These steels exhibit a good combination of formability, strength, ductility, and corrosion resistance, which makes them competitive with other high strength steels and alloys [2].

The refinement of coarse grains of metals and alloys to low dimensions (~100 nm) could improve the mechanical properties of the resulting material [3, 4]. Great efforts have been made to refine coarse grains to nanocrystals using strain-induced grain refinement techniques, including shot peening. Ultrasonic shot peening (USP) was applied to enhance the surface properties of metallic parts, by inducing phase transformation, microstructural refinement, and even nanocrystallization, due to the high-strain rate introduced by high-energy ultrasonic vibration [5–7]. The mechanical properties of steels with low-stacking fault energy, such as austenitic 347 stainless steel, are significantly affected by strain-induced grain refinement and phase transformation. However the degree of plastic deformation on austenitic stainless steel is not clear in previous study. Nevertheless, in this study, this surface treatment induced complex microstructural evolutions such as grain refinement and phase transformation, with an exact percentage of the austenite and *α*′-martensite phases during deformation, which is an expression of the degree of plastic deformation on austenitic stainless steel. In this paper, we try to introduce the application of EBSD techniques to the surface modification of stainless steel upon shot peening. The relationship of processing-microstructure-property was extensively introduced in previous studies.

## 2. Materials and Methods

Materials used in this work are a commercial 347 stainless steel with the chemical composition being C ~ 0.072, Si ~ 0.32, Mn ~ 1.99, P ~ 0.03, S ~ 0.012, Ni ~ 10.1, Cr ~ 18.02, and Nb ~ 0.97 (wt.%). The USP equipment was previously described [5]. Before the treatment, the plate surface was ground to 2000-grit with SiC sand paper. The USP treatment lasted for 5 mins and was performed at room temperature, with an amplitude of 70 *μ*m, frequency of 20 kHz, ball size of 1.5 mm, and a peening area of 2800 mm^2^ in the middle of a plate (110 × 30 × 5 mm^3^).

After the USP treatment, analysis of the surface and cross-sectional region of the specimens was performed using scanning electron microscopy (SEM), electron backscattered diffraction (EBSD), transmission electron microscopy (TEM), and X-ray diffraction (XRD). For the SEM and cross-sectional EBSD measurements, JSM-7800F with Oxford system was used on the surface and cross section of the specimen, with a beam step size of 300 nm. High magnification of the EBSD phase mapping was taken by another EBSD, with a TSL-OIM system installed in a MIRA II LMH FE-SEM, which was used in depth beam scan mode, with a beam step size of 100 nm. TEM measurement was performed on a Philips CM200, operated at 200 kV. XRD analyses of the surface layer were performed using a Rigaku SmartLab-9 X-ray (Japan) diffractometer with Cu K*α* radiation, for 1 hour.

The cross-sectional specimens for EBSD analysis were mechanically polished to 200 *μ*m thickness, followed by electrolytic polishing for 10 s. For the top layer high magnification EBSD analysis and top layer TEM observation, back-thinning processing was used to prepare the top-most surface layer. The top treated surface specimens were thinned to 90 *μ*m, by polishing from the side opposite to the treated side, and then a transparent plastic film was attached to the treated side to prevent etching/polishing during electrolytic polishing. Finally, the TEM specimens were electropolished (Struers TenuPol-5) at room temperature, with an electrolyte mixture of 10% perchloric acid and 90% acetic acid.

## 3. Results and Discussion

**Figure 1:**
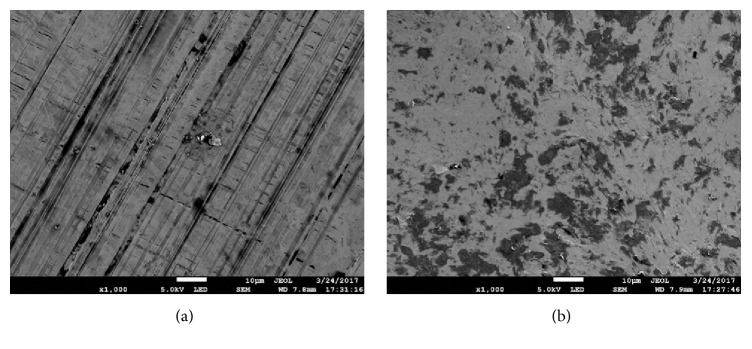
Surface scanning electron microscopy images of (a) untreated and (b) ultrasonic shot peening treated specimens.

Figure 1 shows the surface morphology of (a) untreated and (b) USP-treated specimens, analyzed by SEM. On the untreated specimen, scratches are from different directions, small cracks, and defects seen everywhere; on the USP-treated specimen, the surface modification during peening resulted in a denser and more continuous surface, few defects, and well smoothness.

**Figure 2:**
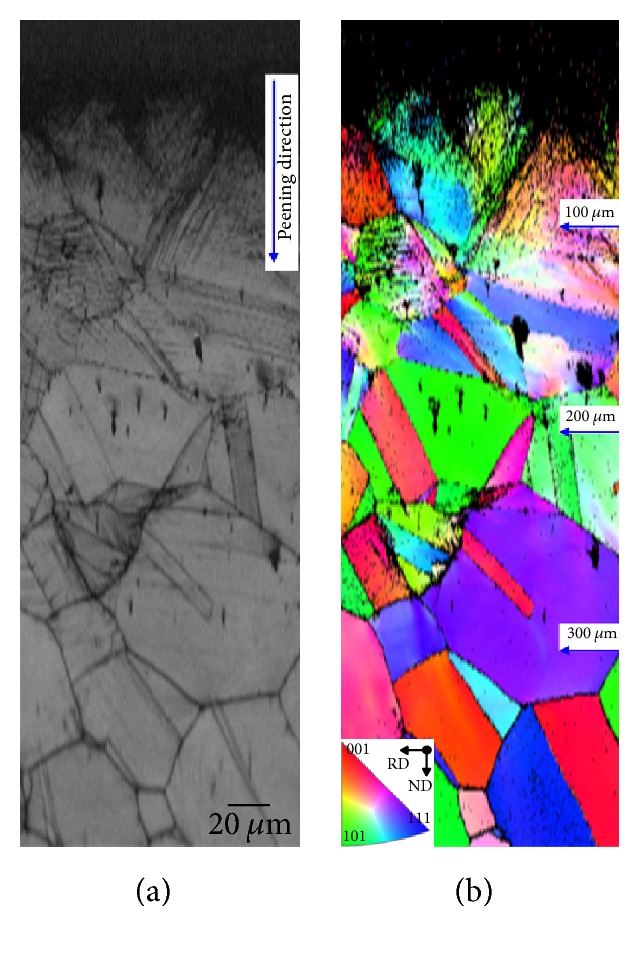
Cross-sectional electron backscatter diffraction (a) image quality and (b) inverse pole figure maps of the ultrasonic shot peening treated specimen.

The effect of plastic deformation was measured via EBSD method form cross section and top view side.  Figure 2 shows cross-sectional (a) image quality (IQ) and (b) inverse pole figure (IPF) maps of the USP-treated specimen. In IQ image, the twin structures are clearly obvious in the inherent grains. Because of USP treatment, in the 0~50 *μ*m depth from the surface, very high-density deformation structures become apparent, and most of them were impossible to read, because the grain size was smaller than the limit of detection (step size of EBSD mapping about 300 nm). High-density deformed slip band structures are obvious in inherent grains in 50~100 *μ*m depth from the surface, whereas low-density slips, with low angle grain boundaries, can be observed in 100~200 *μ*m depth from the surface. The misorientation statistics decrease upon the depth goes down until the out-off effect depth, as well as before plastic deformation matrix. According to a preliminary study, the black dots in the matrix are NbC or M_23_C_6_ particles which are not read during EBSD mapping [8].

**Figure 3:**
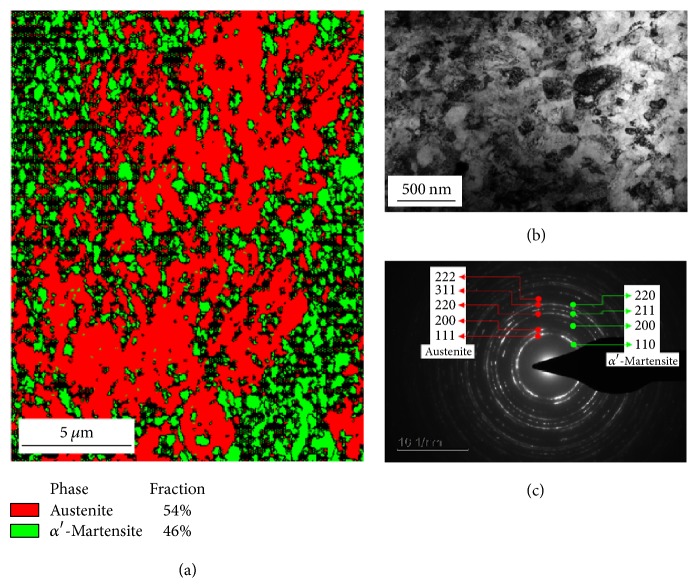
Top-most layer (a) electron backscatter diffraction phase mapping, (b) transmission electron microscopy image, and (c) corresponding selected area diffraction pattern on the top-most layer of the ultrasonic shot peening treated specimen.

Figure 3(a) was taken as high magnification of the EBSD phase mapping method, with a TSL-OIM system installed in a MIRA II LMH FE-SEM, which was used in depth beam scan mode, with a beam step size of 100 nm. The TSL-OIM system can auto-define two phases and give the percentage. The red color indicates the austenite phase, which is a face-centered cubic lattice; green color indicates the *α*′-martensite phase, with body-centered cubic lattice. The grain refinement is obvious, as the two phases maxed each other and grain size was smaller than 100 nm. The percentages of the austenite and *α*′-martensite phases were 54% and 46%, respectively, which are an exact expression of the degree of plastic deformation. Before deformation, the steel matrix was mostly austenite, and the strain caused phase transformation to *α*′-martensite during deformation; therefore the increase in the percentage of second phase is closely related to the degree of deformation, which means that the percentages of the two phases are an exact expression of the degree of plastic deformation on austenitic stainless steels.

**Figure 4:**
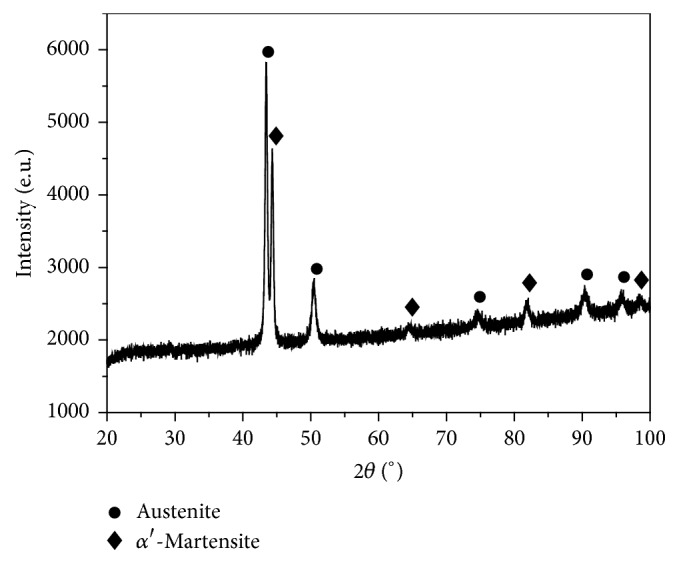
X-ray diffraction profiles of ultrasonic shot peening treated specimen.

Figure 3(b) is a TEM bright field image of the top-most layer of the USP-treated specimen. The refined grains measured about 100 nm, indicating that the USP treatment caused the nanocrystallization of 347 stainless steel. Figure 3(c) is the corresponding selected area diffraction pattern on the top-most layer of the specimen. The ring-like pattern indicates the presence of nanocrystals in this layer, and the two phases of austenite and *α*′-martensite are also clearly obvious (Figure 3(c)). To reconfirm the two-phase structure, XRD analysis was performed on the surface of the USP-treated specimen, and the resulting XRD profiles are shown in Figure 4. The austenite and *α*′-martensite phase peaks are clearly obvious in the XRD profile. However, in this XRD profile analysis, it is difficult to determine the actual percentages of the two phases. In this work, the XRD analysis was used as a qualitative analysis, but the EBSD techniques can be a quantitative analysis.

It is difficult to measure the degree of plastic deformation of various alloys or steels by simple testing. Existing research methods include using stress, microhardness, and grain size to characterize the deformation [9–11]. In this study, the EBSD technique can define the depth and phase percentages of austenitic 347 stainless steel (Figures 2 and 3). The quantitative analysis has important manufacture significance in industrial production. High magnification EBSD phase mapping is an efficient way of determining the percentages of the austenite and *α*′-martensite phases, as shown in this study (54% and 46%, respectively). However, this technique is only appropriate for austenitic steels and for certain strain-induced phase transformation alloys. Furthermore, the percentages of the phases are not the only scale plate to measure deformation. Microstructure deformations including dislocation formation, dislocation density change, grain refinement, twins, and slips, which are not appropriate for EBSD phase mapping. Even though the EBSD technique can exactly express the degree of plastic deformation in the case of austenitic stainless steel, a rational method needs to be developed for the comprehensive evaluation of plastic deformations in other metals or alloys.

## 4. Conclusions

In this study, austenitic 347 stainless steel was USP-treated and investigated by SEM, EBSD, TEM, and XRD. The microstructure evolutions are obvious as grain refinement and phase transformation. EBSD analysis was taken from cross section and top view side. The deformation structures become obvious at about 200 *μ*m depth after USP treatment, and grain size on the treated surface layer was about 100 nm, with two phases: austenite and *α*′-martensite. The percentages of the austenite and *α*′-martensite phases were 54% and 46%, respectively, which are exactly expressing the degree of plastic deformation.
